# Community Assembly Mechanisms of *Populus euphratica* in Northwest China and Their Relationship with Environmental Factors

**DOI:** 10.3390/plants13233283

**Published:** 2024-11-22

**Authors:** Lijun Zhu, Jie Wang, Houji Liu, Juntuan Zhai, Zhijun Li

**Affiliations:** 1 Xinjiang Production & Construction Corps Key Laboratory of Protection and Utilization of Biological Resources in Tarim Basin, Alar 843300, China; 15393470171@163.com (L.Z.); wjie2023418@163.com (J.W.); liuhj2023163@163.com (H.L.); zhaijuntuan2022@163.com (J.Z.); 2 College of Life Science and Technology, Tarim University, Research Center of Populus Euphratica, Alar 843300, China

**Keywords:** plant community, species diversity, phylogenetic diversity, competitive exclusion, *Populus euphratica*

## Abstract

*Populus euphratica* is a key community-building species in the desert riparian forests of Northwest China, exhibiting exceptional resistance to stress and playing a vital role in soil and water conservation as well as maintaining ecological balance in arid regions. To investigate the ecological processes underlying the composition of *P. euphratica* communities and to identify their community construction mechanisms, this study analyses the species diversity and phylogenetic diversity of 58 *P. euphratica* communities, exploring their assembly processes and key influencing factors. This research aims to elucidate the relationship between community structure from the perspective of species evolution and analyse the construction mechanisms of *P. euphratica* communities across different clusters in arid environments. The results show that the species diversity of *P. euphratica* clusters in Northwest China is relatively low, and a significant correlation is noted with phylogenetic diversity (PD). The Shannon–Wiener and Margalef indices exhibit similar trends, whereas Simpson’s index show the opposite trends. Pielou’s index range from 0.7 to 0.85. Notably, the PD and species diversity of the *P. euphratica*–*Haloxylon ammodendron* association group (Group 4) is significantly higher (*p* < 0.05) compared to that of the other groups. Additionally, net relatedness index (NRI) and nearest taxon index (NTI) peaked in the *P. euphratica*–*H. ammodendron* association group (Group 4) and the *Populus pruinosa*–*Tamarix ramosissima*–*Phragmites australis* association group (Group 1) (*p* < 0.05). A Pearson correlation analysis indicated that PD was significantly positively correlated with Margalef’s index, Shannon–Wiener’s index, and Pielou’s index, but was significantly negatively correlated with Simpson’s index, while also being associated with environmental factors. Key factors influencing the diversity of *P. euphratica* communities in Northwest China include total phosphorus, pH, soil moisture content, total potassium, the mean temperature of the coldest quarter, precipitation of the wettest month, and precipitation seasonality. Soil factors primarily affected the Pielou and Simpson indices of species diversity, whereas climatic factors mainly influenced the Margalef and Shannon–Wiener indices. PD and structure were mainly influenced by climatic factors. The combined effects of soil and climatic factors play a crucial role in sustaining the diversity and ecological adaptation of these plant communities. In summary, *P. euphratica* communities may exhibit a significant ecological niche conservation in response to environmental changes, and competitive exclusion might be the primary process shaping community structure. Climatic factors were shown to be important regulators of community diversity and phylogenetic structure.

## 1. Introduction

Forest ecosystems are among the most biologically diverse ecosystems on Earth and play an important role in maintaining ecological balance [1]. In recent years, influenced by climate warming, extreme weather events, and human activities, the species composition and species diversity of forest communities have undergone significant changes, posing a threat to ecosystem functions [2,3]. Biodiversity is an important material foundation for maintaining ecosystem functions. Numerous studies have demonstrated that biodiversity can enhance ecosystem functioning [4,5]. Biodiversity research includes two major indicators: species diversity and phylogenetic diversity (PD). Species diversity reveals spatiotemporal variations through species count and abundance, having a positive impact on ecosystem functioning [6,7,8,9]. PD reflects community diversity and maintenance mechanisms through evolutionary relationships [10,11]. PD is highly correlated with species diversity [12,13]. For instance, Qian et al. [14] research found that PD in tropical South America is higher than that in tropical Africa, with the results corresponding to observed changes in species richness.

Species coexistence plays a crucial role in ecosystem functioning. Diverse species communities typically enhance ecosystem stability, productivity, and resistance to disturbance. Understanding the mechanisms of plant community assembly is essential to explain species coexistence. Current research on community assembly mechanisms is primarily based on two frameworks: ecological niche theory and neutral theory. Ecological niche theory suggests that community assembly is a deterministic filtering process, wherein habitat filtering and competitive exclusion regulate species diversity and maintain community stability [15,16]. On the contrary, neutral theory assumes that all species at the same trophic level are functionally equivalent, suggesting that community dynamics are governed by stochastic processes, with dispersal limitation playing a deterministic role in shaping community structure [17]. When habitat filtering is dominant, species tend to be closely related to one another, leading to an aggregated phylogenetic structure. Conversely, when competitive exclusion prevails, species are distantly related, resulting in phylogenetic overdispersion. Recent studies indicated that environmental filtering, competitive exclusion, and dispersal limitation all contribute to community assembly, but their relative importance varies depending on environmental conditions [18,19]. Webb [20] was the first to apply phylogenetic methods to investigate community assembly mechanisms in tropical rainforest communities, introducing phylogenetic structure indicators. Silva and Batalha’s [21] research on savanna plants in southern Baja found that Cerrado plant communities are primarily shaped by competitive exclusion, as evidenced by patterns of phylogenetic overdispersion.

The process of plant community construction is influenced by a variety of environmental factors, with climate and soil conditions receiving significant attention [22,23,24]. Research has shown a strong relationship between plant species richness in arid regions and temperature and precipitation [25,26]. A study of desert grasslands in Northwest China identified soil factors as key drivers of plant diversity and productivity [27]. In the semi-arid region of the western Loess Plateau of China, precipitation has been suggested as the primary factor influencing change in shrubland type and diversity [28]. Environmental factors, as crucial drivers of community assembly, shape community structure and function by influencing species distribution, ecological niche differentiation, and the dynamic succession of communities. At the same time, environmental factors contribute to community diversity and stability. Climatic influences plant growth rates and adaptative strategies, while soil alters plant physiological traits and competitive dynamics. Therefore, they shape the complex structure and diversity of communities.

*Populus* is a genus within the Salicaceae family, encompassing over 100 species that are widely distributed across Europe, Asia, and North America. Among these, *Populus euphratica* is recognised as one of the most ancient and broadly distributed species within the genus. Its primary distribution includes the northwestern regions of China, as well as Uzbekistan, Kazakhstan, Kyrgyzstan, and other neighbouring countries [29]. It is the dominant species in desert riparian forests and a rare forest species naturally found in desert regions [30]. In China, the largest and most concentrated distribution of *P. euphratica* is the Tarim Basin. *P*. *euphratica*, renowned for its exceptional drought tolerance, cold hardiness, and heat resistance, plays a crucial role in preventing wind erosion, stabilising sand dunes, conserving water resources, supporting agricultural and pastoral systems, and maintaining the ecological balance of arid regions [31,32]. In the past, research on *P. euphratica* has mainly focused on the species’ stress resistance [33,34] and the impact of ecological water conveyance on species diversity within *P. euphratica* communities [35,36]. Studies on species distribution and competition within *P. euphratica* communities have focused on species composition and community structure. For instance, Zhou et al.’s [37] investigation into the community diversity of *P. euphratica* forests in the Ejina Oasis in northern China revealed relatively low biodiversity in the study area, but high community diversity across different habitats. Similarly, the study by Han et al. [38] on desert riparian forests at the source of the Tahe River in northwestern China indicated low species diversity, with interspecific competition and habitat heterogeneity identified as critical factors influencing species distribution and coexistence in desert riparian ecosystems. In addition, soil and climatic factors are critical in influencing *P. euphratica* communities and provide important guidance for the conservation and restoration of desert riparian vegetation. Soil salinity significantly affects vegetation cover, with high salinity conditions reducing the germination rate of *P. euphratica* seeds [39]. By contrast, rising groundwater levels can lower soil salinity and increase humidity, thereby enhancing plant diversity. Studies have suggested that global warming accelerates evapotranspiration and decreases soil moisture, potentially leading to forest degradation [40]. Therefore, an in-depth investigation of the effects of climatic and soil factors on *P. euphratica* communities is essential for the conservation of ecosystem in the region.

Previous studies on *P. euphratica* communities primarily focused on species diversity indices and genus ratios to reflect species richness and community characteristics. However, there remains a significant research gap in utilising species phylogenetic information to explore community maintenance mechanism from an evolutionary perspective. Investigating the species diversity and phylogeny of *P. euphratica* communities is not only crucial for the restoration of forest vegetation in arid zones, but also provides valuable insights for biodiversity conservation efforts. This study, through field investigations of *P*. *euphratica* communities, data collection, and soil sample analysis, examines species diversity and PD from the perspective of community ecology. It further explores the relationships between these diversity characteristics and environmental factors. This research aims to elucidate the assembly mechanisms of *P. euphratica* communities and identify their key influencing factors, providing a scientific basis for the conservation and sustainable management of desert ecosystems. Specifically, this study addresses the following research questions: (1) Are there differences in plant species diversity and PD among various *P. euphratica* communities in the northwest region in China? (2) What are the underlying causes of these differences in community structure? (3) What are the community structure mechanisms in different *P. euphratica* communities in Northwest China? (4) What environmental factors influence the PD and structure of *P. euphratica* communities?

## 2. Results

### 2.1. Taxonomic Composition of Plant Species and Phylogenetic Tree Construction

**Figure 1:**
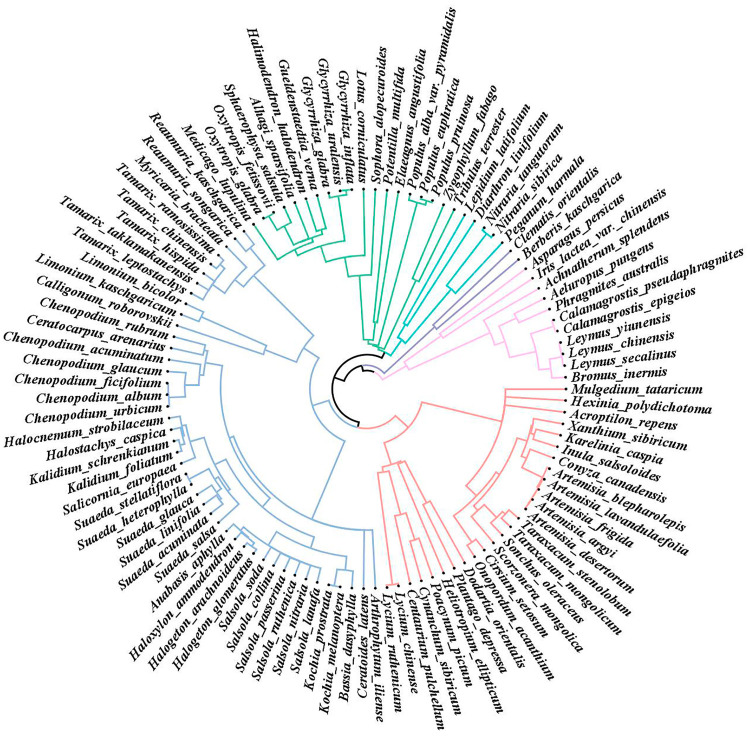
Phylogenetic tree of poplar community.

A total of 24 families, 68 genera, and 107 plant species were recorded across 58 sampling sites (Supplementary Material Table S1). Among these, Amaranthaceae had the highest species richness, with 15 genera and 33 species, accounting for 30.84% of the total species surveyed. This was followed by Asteraceae, which comprised 16.82% of the species. Several families, including Plantaginaceae, Elaeagnaceae, Polygonaceae, Gentianaceae, Ranunculaceae, Rosaceae, Thymelaeaceae, Brassicaceae, Heliotropiaceae, Asparagaceae, Mazaceae, Berberidaceae, and Iridaceae, were represented by only one species each. The phylogenetic tree of the study area is based on biological systems and divided into six major taxa using the evolutionary distance between species (Figure 1).

(1) The first group comprised 11 plant species from Poaceae, Iridaceae, and Asparagaceae.
(2) The second group included two species from Berberidaceae and Ranunculaceae.
(3) The third group consisted of five species from Nitrariaceae, Thymelaeaceae, and Brassicaceae.
(4) The fourth group was composed of 19 species from Zygophyllaceae, Salicaceae, Elaeagnaceae, Rosaceae, and Fabaceae.
(5) The fifth group consisted of 44 species from Tamaricaceae, Plumbaginaceae, Polygonaceae, and Amaranthaceae.
(6) The sixth group included 26 species from Asteraceae, Mazaceae, Plantaginaceae Heliotropiaceae, Apocynaceae, Gentianaceae, and Solanaceae.

### 2.2. Community Types and Their Species Composition

**Figure 2:**
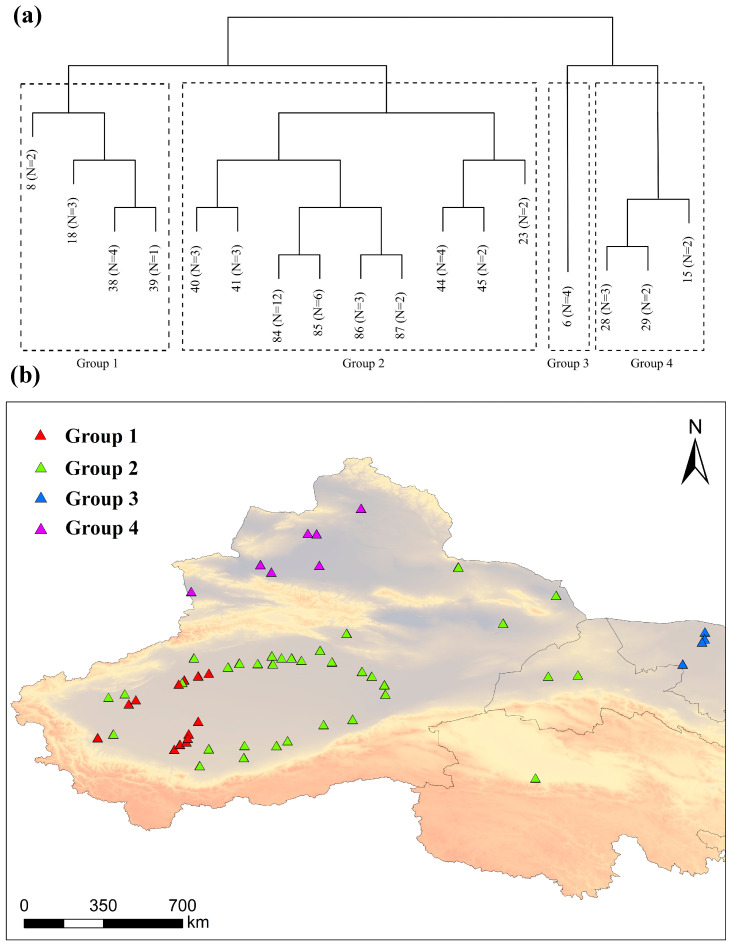
(**a**) Division of *Populus euphratica* community types. (**b**) Sample point distribution maps for the four cluster groups.

The 58 sampling sites were classified into four clusters via a combination of Twinspan and DCA ordination (Figure 2). These four cluster groups were as follows: *P. pruinosa–Tamarix ramosissima–Phragmites australis* association group, *P. euphratica–T. ramosissima–P. australis* association group, *P. euphratica–H. ammodendron–Sophora alopecuroides* association group, and *P. euphratica*–*H. ammodendron* association group.

Group 1: *P. pruinosa*–*T. ramosissima*–*P. australis* association group. This group included 13 sample plots. The dominant species in the tree layer was *P. pruinosa*, with associated species such as *P. euphratica* and *Populus alba var. pyramidalis*. In the shrub layer, the dominant species was *T. ramosissima*, accompanied by *Lycium ruthenicum*, *Halimodendron halodendron*, *Poacynum pictum*, and others. The dominant species in the herbaceous layer was *P. australis*, with accompanying species including *Glycyrrhiza inflata*, *Aeluropus pungens*, *Calamagrostis pseudaphragmites*, *Sophora alopecuroides*, and *Leymus secalinus*.

Group 2: *P. euphratica*–*T. ramosissima*–*P. australis* association group. This group included 34 sample plots. The dominant species in the tree layer was *P. euphratica*, with associated species such as *P. pruinosa* and *Elaeagnus angustifolia*. In the shrub layer, *T. ramosissima* was the dominant species, accompanied by *Alhagi sparsifolia*, *L*. *ruthenicum*, *Tamarix hispida*, *P. pictum*, *H. halodendron*, *Tamarix taklamakanensis*, and *H. ammodendron*. The dominant species in the herbaceous layer was *P. australis*, with accompanying species including *Karelinia caspia*, *G. inflata*, *Salsola ruthenica*, *Calamagrostis epigeios*, *Cynanchum sibiricum*, and *A. pungens*.

Group 3: *P. euphratica*–*H. ammodendron*–*S. alopecuroides* association group. This group included 4 sample plots. The dominant species in the tree layer was *P. euphratica*, while the dominant species in the shrub layer was *H. ammodendron*, accompanied by species such as *T. ramosissima* and *Tamarix leptostachys*. In the herbaceous layer, *S. alopecuroides* was the dominant species, with companion species including *Peganum harmala*, *L. secalinus*, and *K. caspia*.

Group 4: *P. euphratica*–*H. ammodendron* association group. This group included seven sample plots. The dominant species in the tree layer was *P. euphratica*, while *H. ammodendron* dominated the shrub layer, with associated species such as *Nitraria sibirica*, *H. halodendron*, *Ceratoides latens*, *A. sparsifolia*, *Artemisia blepharolepis*, and *T. ramosissima*. The herbaceous layer was composed of species such as *Suaeda stellatiflora*, *S. alopecuroides*, *Achnatherum splendens*, and *Salsola nitraria*.

### 2.3. Plant Species Diversity in Different Community Types

**Figure 3:**
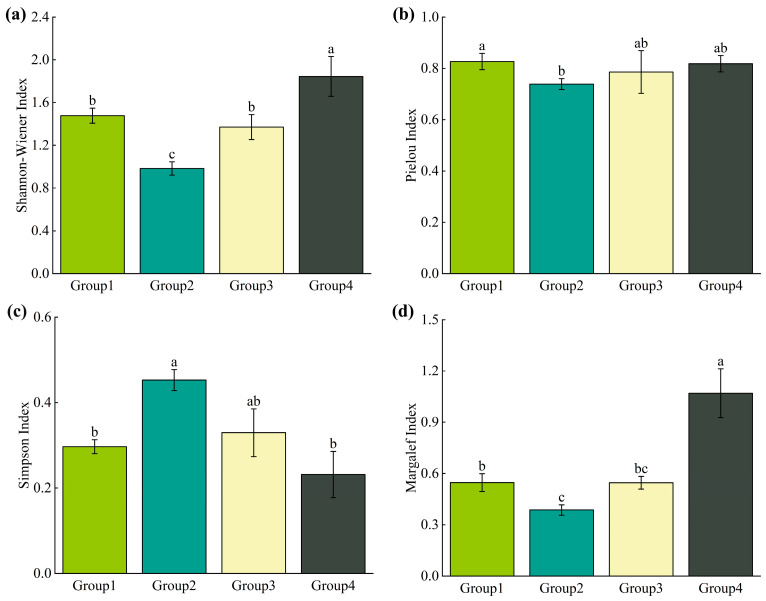
(**a**) Variation in Shannon-–Wiener Diversity Index in different communities (*p* < 0.05). (**b**) Variation in Pielou Evenness Index in different communities (*p* < 0.05). (**c**) Variation in Simpson Dominance Index in different communities (*p* < 0.05). (**d**) Variation in Margalef Richness Index in different communities (*p* < 0.05).

The results presented in Figure 3 show that both the Shannon–Wiener and Margalef indices followed similar trends, initially decreasing before subsequently increasing. The *P. euphratica*–*H. ammodendron* association group (Group 4) showed the highest values for both indices (1.844 and 1.070, respectively), significantly exceeding those of the other community types. In descending order, the indices were as follows: *P. pruinosa*–*T. ramosissima*–*P. australis* association group (Group 1), *P. euphratica*–*H. ammodendron*–S. alopecuroides association group (Group 3), and *P. euphratica*–*T. ramosissima*–*P. australis* association group (Group 2). By contrast, the Simpson index displayed an opposite trend compared with the Shannon–Wiener and Margalef’s indices. The Simpson index for the *P. euphratica*–*T. ramosissima*–*P. australis* association group (Group 2; 0.453) was significantly higher than that for the *P. pruinosa*–*T. ramosissima*–*P. australis* association group (Group 1; 0.297) and the *P. euphratica*–*H. ammodendron* association group (Group 4; 0.232). The Pielou index, which reflected the evenness of species distribution within communities, ranged from 0.7 to 0.85 across the four groups. The *P. pruinosa*–*T. ramosissima*–*P. australis* association group (Group 1) had the highest evenness index, significantly surpassing that of the *P. euphratica*–*T. ramosissima*–*P. australis* association group (Group 2). Overall, the results suggest that the *P. euphratica*–*H. ammodendron* association group (Group 4) had the highest species diversity, whereas the *P. pruinosa*–*T. ramosissima*–*P. australis* association group (Group 1) exhibited the most evenly distributed species composition.

### 2.4. PD and Phylogenetic Structure

#### 2.4.1. PD of Different Community Types

**Figure 4:**
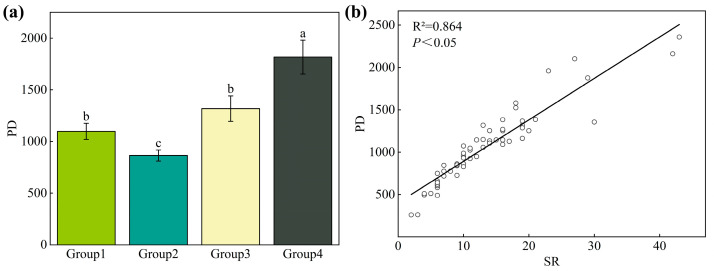
(**a**) Variation in phylogenetic diversity among different community types (*p* < 0.05). (**b**) Relationship between phylogenetic diversity and species richness.

As shown in Figure 4a, the *P. euphratica*–*H. ammodendron* association group (Group 4) exhibited the highest PD value (1816.44), which was significantly higher (*p* < 0.05) than that of the other community groups. The PD values for the other groups, in descending order, were as follows: *P. euphratica*–*H. ammodendron*–*S. alopecuroides* association group (Group 3; 1317.66), *P. pruinosa*–*T. ramosissima*–*P. australis* association group (Group 1; 1097.36), and *P. euphratica*–*T. ramosissima*–*P. australis* association group (Group 2; 864.12). Additionally, a significantly positive correlation (*p* < 0.05) was observed between the PD and SR of the plant communities (Figure 4b).

#### 2.4.2. Phylogenetic Structure of Different Community Types

**Figure 5:**
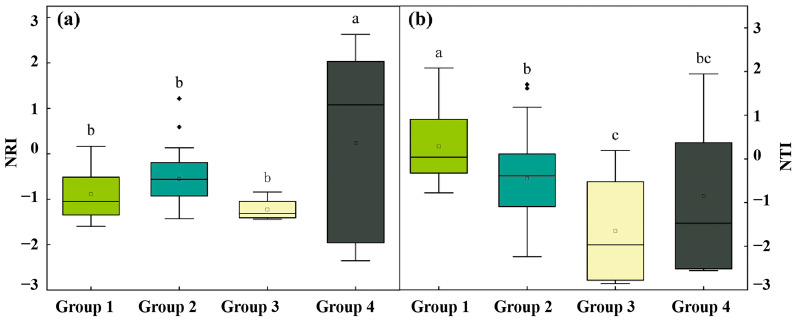
(**a**) Variation in NRI among different community types (*p* < 0.05). (**b**) Variation in NTI among different community types (*p* < 0.05).

The NRI reached its highest value in the *P. euphratica*–*H. ammodendron* association group (Group 4) and was significantly different from the three other groups (*p* < 0.05). The *P. pruinosa*–*T. ramosissima*–*P. australis* association group (Group 1), *P. euphratica*–*T. ramosissima*–*P. australis* association group (Group 2), and *P. euphratica*–*H. ammodendron*–*S. alopecuroides* association group (Group 3) did not show significant differences from one another (Figure 5a). The NTI reached its maximum in the *P. pruinosa*–*T. ramosissima*–*P. australis* association group (Group 1) and was significantly different from the three other groups (*p* < 0.05). We found no significant difference in NTI between the *P. euphratica*–*T. ramosissima*–*P. australis* association group (Group 2) and *P. euphratica*–*H. ammodendron* association group (Group 4) (Figure 5b).

**Figure 6:**
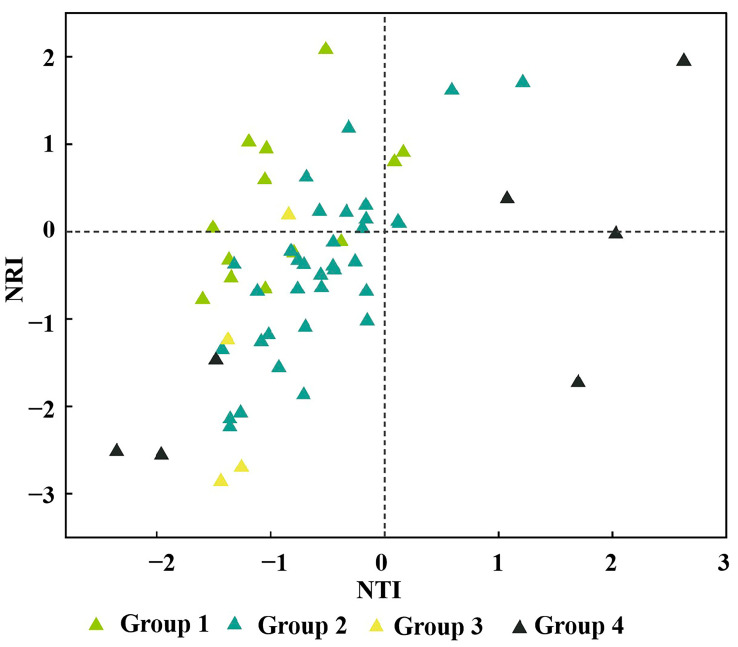
Distribution of phylogenetic indices for different community types.

Among the 58 sampled sites, 8 exhibited NRI and NTI values greater than 0, indicating an aggregated phylogenetic structure, where the community consisted of closely related species. By contrast, 35 sampled sites had NRI and NTI values less than 0, suggesting a dispersed phylogenetic structure, with the community composed of distantly related species. The remaining 15 sampled sites showed inconsistent positive and negative NRI and NTI values, making it difficult to determine the phylogenetic structure of these community (Figure 6). In the *P. pruinosa*–*T. ramosissima*–*P. australis* association group (Group 1), two sampled sites exhibited clustered phylogenetic structures, six sampled sites displayed dispersed phylogenetic structures, and five sampled sites could not be categorised. The *P. euphratica*–*T. ramosissima*–*P. australis* association group (Group 2) contained four sampled sites with clustered phylogenetic structures, twenty-three sampled sites with dispersed phylogenetic structures, and seven sampled sites that could not be judged. For the *P. euphratica*–*H. ammodendron*–*S. alopecuroides* association group (Group 3), no samples were phylogenetically clustered, three had dispersed phylogenetic structures, and one sampled site could not be categorised. Lastly, in the *P. euphratica*–*H. ammodendron* association group (Group 4), two sampled sites exhibited clustered phylogenetic structures, three sampled sites showed dispersed phylogenetic structures, and two could not be determined.

By performing *t*-tests for NRI and NTI across different cluster groups (Table 1), no significant difference was found between NRI and 0 in the *P. euphratica*–*H. ammodendron* association group (Group 4; *p* > 0.05). Similarly, NTI showed no significant difference from 0 in the *P. pruinosa*–*T. ramosissima*–*P. australis* association group (Group 1), *P. euphratica*–*H. ammodendron*–*S. alopecuroides* association group (Group 3), and *P. euphratica*–*H. ammodendron* association group (Group 4; *p* > 0.05). However, the NRI means for the *P. pruinosa*–*T. ramosissima*–*P. australis* association group (Group 1), *P. euphratica*–*T. ramosissima*–*P. australis* association group (Group 2), and *P. euphratica*–*H. ammodendron*–*S. alopecuroides* association group (Group 3) were significantly less than 0 at the 95% confidence interval (*p* < 0.05), indicating that the phylogenetic structure exhibited divergence. Additionally, the NTI mean for the *P. euphratica*–*T. ramosissima*–*P. australis* association group (Group 2) was significantly less than 0 (*p* < 0.05) at the 95% confidence interval, suggesting PD. These results indicate that species within these communities were distantly related, with community assembly predominantly driven by competitive exclusion.

### 2.5. Analysis of Factors Influencing P. euphratica Communities

#### 2.5.1. Selection of Environmental Factors

**Figure 7:**
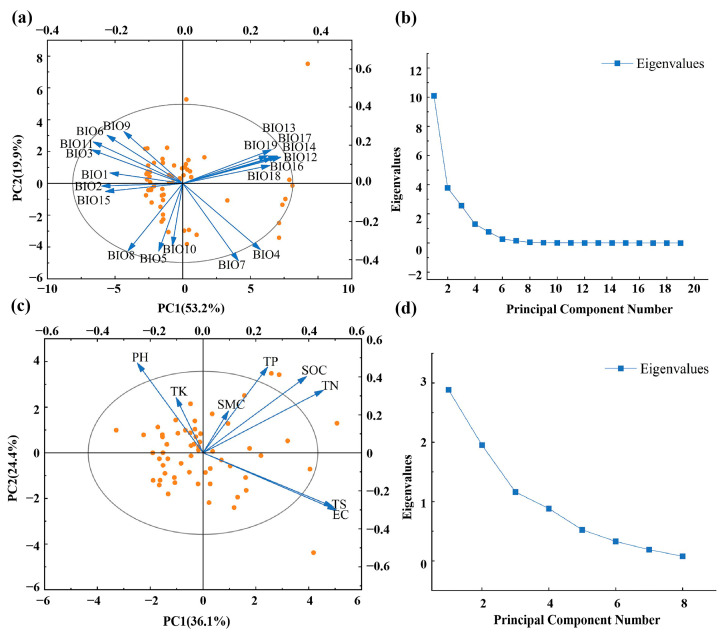
Principal component analysis of climate and soil factors. (**a**) PCA of climate factors; (**b**) scree plot of climate factor PCA; (**c**) PCA of soil factors (SMC: soil moisture content; pH; EC: electrical conductivity; SOC: soil organic carbon; TS: total salt; TK: total potassium; TN: total nitrogen; TP: total phosphorus.); (**d**) scree plot of soil factor PCA.

PCA of the 19 climate factors (Supplementary Material Table S2) revealed that the first, second, and third principal components accounted for 53.17%, 19.89%, and 13.50% of the original information, respectively. Together, these three components explained over 80% of the original variance. After eliminating covariance, three climate factors, namely BIO11 (mean temperature of the coldest quarter), BIO13 (precipitation of the wettest month), and BIO15 (precipitation seasonality), were selected as representative indicators (Figure 7a,b).

PCA of the eight soil factors revealed that the first four principal components accounted for 86% of the original variance. The first, second, third, and fourth components explained 36.09%, 24.40%, 14.49%, and 11.02% of the original variance, respectively. After eliminating covariance, four soil factors, namely SMC (soil moisture content), TK (total potassium), pH, and TP (total phosphorus), were selected as representative indicators (Figure 7c,d).

#### 2.5.2. Correlations Among Species Diversity, PD, and Environmental Factors

**Figure 8:**
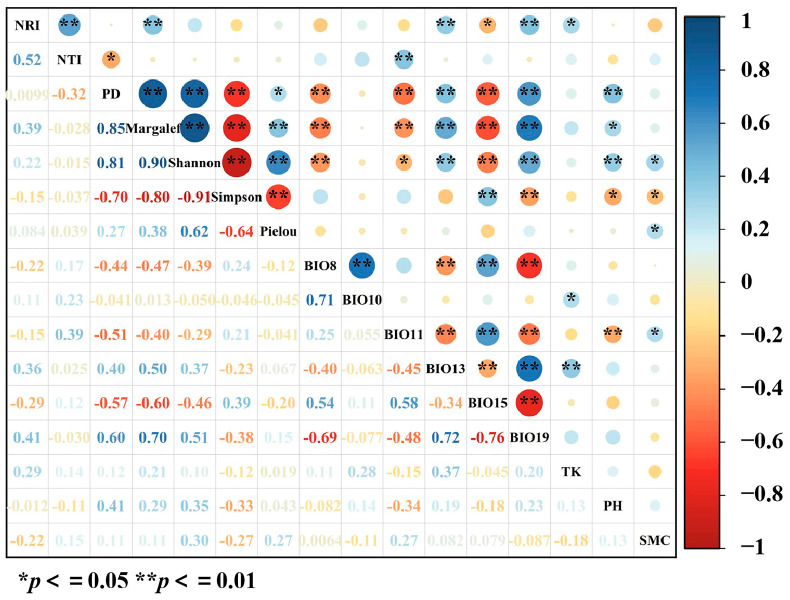
Correlation between species diversity, phylogenetic diversity, and environmental factors.

We found a significant positive correlation among the Margalef index, Shannon–Wiener index, Pielou index, and PD. NRI exhibited a significant positive correlation with NTI and the Margalef index. By contrast, Simpson’s index showed a significant negative correlation with the Margalef index, Shannon–Wiener index, Pielou index, and PD, whereas NTI was negatively correlated with PD (Figure 8).

BIO13 and PH exhibited significant positive correlations with PD, the Margalef index, and the Shannon–Wiener index, whereas BIO11 and BIO15 were significantly negatively correlated with these indices. BIO13 and TK were significantly positively correlated with NRI, BIO11 was significantly positively correlated with NTI, and BIO15 showed a significant negative correlation with NRI. Additionally, TP was significantly positively correlated with the Margalef and Shannon indices, SMC was positively correlated with the Pielou and Shannon indices, and BIO15 was positively correlated with the Simpson index, whereas PH and SMC were significantly negatively correlated with the Simpson index (Figure 8).

#### 2.5.3. Climate and Soil Explanations for Species Diversity and PD

**Figure 9:**
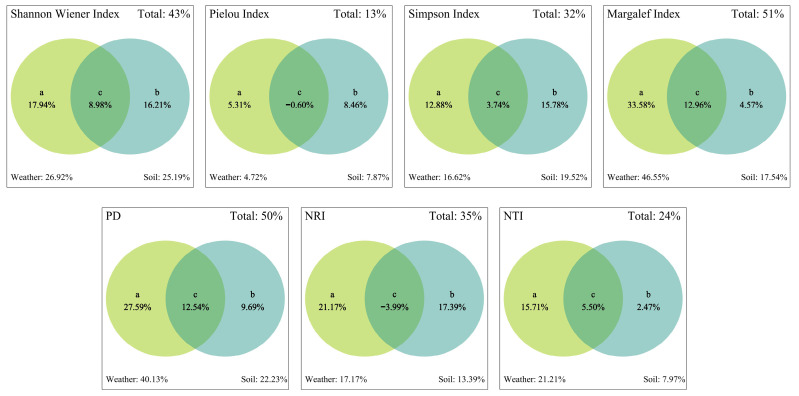
Variance decomposition of species diversity and phylogenetic diversity with climate and soil (a: The explanatory power of climate alone for a specific indicator. b: The explanatory power of soil alone for a specific indicator. c: The combined explanatory power of soil and climate for a specific indicator).

As shown in Figure 9, climatic factors alone had the highest explanatory power, accounting for 33.58%, 27.59%, and 15.71% of the variation in the Margalef index, PD, and NTI, respectively. By contrast, soil factors alone explained 4.57%, 9.69%, and 2.47% of the variation in the Margalef index, PD, and NTI, respectively. The combined explanatory power of climate and soil factors for the Margalef index, PD, and NTI was 12.96%, 12.54%, and 5.50%, respectively, which exceeded that of soil factors alone. Soil and climate factors alone showed similar explanatory power for the Shannon–Wiener index, Pielou index, Simpson index, and NRI. Notably, soil factors had a greater influence on the Pielou and Simpson indices than climate factors or their combined effect, whereas climate factors had a stronger explanatory power for the Shannon–Wiener index and NRI compared with soil factors and their joint influence. These results indicate that the Pielou and Simpson indices of species diversity were primarily influenced by soil factors, whereas the Margalef and Shannon–Wiener indices were predominantly affected by climate factors. PD and structure were mainly governed by climatic factors.

## 3. Discussion

### 3.1. Diversity Variation and Correlations Across Different Community Types

In the context of climate warming and the gradual loss of biodiversity, a comprehensive consideration of species diversity and PD within plant communities can offer effective conservation strategies for arid and semi-arid ecosystems. Classifying plant community types is a complex task due to the diversity of ecosystems and the numerous environmental factors to which they are subjected. Currently, the combination of species importance value or dominance with the Twinspan classification method has been widely adopted in plant community research. For instance, in a study of mixed forests in Jingu Forest, Twinspan was used to classify the forests into seven distinct types [41]. Similarly, in research conducted on the lower reaches of the Tawar River in Pakistan, Twinspan was employed to classify different vegetation types and identify various communities, providing essential data for regional ecological assessments [42]. In this study, we adopted these established methods, classifying the *P. euphratica* community into four distinct groups based on cluster analysis of the importance values across different strata within the community. The results reveal that the *P. euphratica*–*H. ammodendron* association group (Group 4) exhibited the richest species diversity, while the *P. euphratica*–*T. ramosissima*–*P. australis* association group (Group 2) showed the simplest species diversity (Figure 3). In terms of community composition, the *P. euphratica*–*H. ammodendron* association group (Group 4) was primarily distributed in northern Xinjiang, where annual precipitation ranges between 150 and 200 mm, the average temperature is between −4 °C and 9 °C, and relatively favourable hydrothermal conditions promote rich vegetation. By contrast, the *P. euphratica*–*T. ramosissima*–*P. australis* association group (Group 2) was predominantly found in the Tarim Basin, an extremely arid region characterised by extensive desert landscapes, minimal annual precipitation, high evaporation rates, and harsh environmental conditions. Overall, the species diversity index for *P. euphratica* communities in Northwest China was low, with species composition at most sample sites ranging between 2 and 15 species, reflecting a simple community structure. This result indicates the fragile nature of the desert riparian in Northwest China, where few plant species can survive. These findings align with the previous studies on plant diversity in the Tarim River Basin [43,44] and the Black River Basin [45].

This study reveals notable differences in species composition and richness across the various community types. The *P. euphratica*–*H. ammodendron* association group (Group 4) exhibited relatively high species diversity and richness, with its species composition encompassing nearly all the species found in the three other clusters. Additionally, the PD index of this group was significantly higher than that of the three other clusters, indicating a broad genetic reservoir and enhanced adaptability in terms of evolutionary history and ecological resilience. By contrast, the *P. euphratica*–*T. ramosissima*–*P. australis* association group (Group 2) displayed a simpler species composition and correspondingly low PD, suggesting limitations in the evolutionary relationships and ecological adaptive of its species. This finding underscored the positive correlation between PD and species diversity within communities [46,47]. Communities with high PD were found to enhance primary community stability and exhibit greater evolutionary potential and adaptive capacity, particularly in response to external stressors such as future climate change [48]. Consequently, conservation efforts should prioritise areas where the *P. euphratica*–*H. ammodendron* association group (Group 4) is distributed, given its significant ecological value and adaptive potential.

Furthermore, the results demonstrate that PD was significantly positively correlated (*p* < 0.05) with species richness, the Margalef index, the Shannon–Wiener index, and the Pielou index, but significantly negatively correlated with the Simpson index (Figure 8). These findings suggest that PD was not only influenced by species richness, but was also closely associated with species evenness and the overall diversity of the community [49]. In a study of plant communities in the Dongling Mountain, Ma et al. [50] indicated that the Shannon–Wiener index is highly sensitive to rare species. In species-sparse communities, the phylogenetic structure of the community was dispersed. Additionally, our study reveals that NRI was significantly positively correlated with NTI and the Margalef index, indicating a tendency for the community’s phylogenetic structure to become clustered with increasing species richness. By contrast, no significant correlations were observed among NTI, NRI, and the Shannon–Wiener index, Simpson index, or Pielou index, which may be attributed to the communities in the study area being at an early stage of ecological succession. At this stage, the evolutionary relationships between species have not fully emerged, and the phylogenetic structure of the community is still developing, resulting in the lack of significant relationships with diversity and evenness indices.

### 3.2. Phylogenetic Structure Reveals Aggregation Patterns in Different Community Types

Investigating the phylogenetic relationships of species allows us to comprehend the distribution and aggregation patterns of species across different community types, providing crucial insights into the processes driving community assembly. A significant positive correlation (*p* < 0.05) was found between PD and species richness (Figure 4), suggesting that *P. euphratica* communities in Northwest China have undergone an extended evolutionary process and exhibit ecological niche conservation in response to environmental changes. Similar conclusions have been drawn from previous studies on plant communities in the Gurbantunggut Desert [51] and the Taihang Mountains [52]. Moreover, significant differences in NRI and NTI were identified across the various cluster groups (Figure 5). The NRI reached its highest value in the *P. euphratica*–*H. ammodendron* association group (Group 4), indicating strong species associations and close phylogenetic relationships within the community. By contrast, NTI was highest in the *P. pruinosa*–*T. ramosissima*–*P. australis* association group (Group 1), suggesting that species within this group were closely related, with a tendency toward small-scale aggregation. The contrasting patterns of NRI and NTI across clusters reflected the spatial distribution and species aggregation within the community, likely influenced by environmental factors and successional stages [53,54].

The analysis of the phylogenetic structure of 58 *P. euphratica* community samples revealed significant heterogeneity: 8 sample sites exhibited phylogenetic aggregation, 35 sample sites had dispersed phylogenetic structure, and 15 sample sites could not be judged (Figure 6). These differences highlight the diversity and complexity of species composition within the *P. euphratica* communities, suggesting that the phylogenetic structure of different plant communities demonstrated distinct patterns of aggregation and dispersion based on kinship [55,56]. The phylogenetic structure of the communities was shaped by the combined effects of ecological niche differentiation, competitive exclusion, and environmental filtering during community assembly. Most of the samples from the four flora groups exhibited phylogenetic dispersion, and only a few showed phylogenetic aggregation. Thus, the species composition of different flora groups varied in terms of evolutionary relationships, with community assembly processes largely influenced by competitive exclusion. Competition among species played a crucial role in shaping community structure and species diversity. Research on the plant communities of the Huangshan Mountain showed that competitive exclusion was prominent in deciduous broadleaf forests, while environmental filtering was the dominant force in evergreen broadleaf and mixed coniferous–broadleaf forests [57]. Notably, competitive exclusion may eliminate distantly related species with similar ecological niches but low competitive abilities, resulting in an aggregated phylogenetic structure [58]. In the Iberian Atlantic steppe, weak ecological niche differentiation among species allowed for competitive exclusion to drive phylogenetic aggregation [59]. Conversely, a study of communities in New Jersey, USA, found that phylogenetic dispersion was driven by the survival of distantly related species, with species loss primarily influenced by stochastic processes rather than competitive exclusion [60]. Thus, balancing deterministic and stochastic processes will be a key focus for future research in this study area.

### 3.3. Environmental Factors Influencing Species Diversity and PD in P. euphratica Communities

Soil nutrients are crucial for plant growth and development, significantly influencing the diversity of plant communities. TP affects the growth rate of above- and below-ground plants parts, with a particularly marked impact on the above-ground portions [61]. Moreover, under limited soil moisture, phosphorus enhances plant photosynthesis, water utilisation, and the capacity to survive drought [62]. Excessive soil pH can deteriorate soil structure and hinder nutrient uptake, potentially resulting in species loss and reduced biodiversity [63]. Species diversity and PD in *P. euphratica* communities in Northwest China were significantly correlated with TP, pH, and SMC, suggesting that these soil factors collectively shaped the composition and structure of plant communities. Similar findings have been reported in studies of plant diversity in China’s Loess Hills Plateau [64], the arid and semi-arid region of central South Africa [65], and the Gurbantunggut Desert [51]. Additionally, NRI was found to be significantly correlated with TK. This may be due, in part, to the distribution of *P. euphratica* communities in arid and semi-arid regions, where vegetation is subject to extreme drought. Potassium enhances plant dry matter accumulation, increasing root surface area and improving water absorption, which helps plants cope with drought [66]. Furthermore, as a key nutrient for plant growth, potassium influences species adaptability and competitiveness, thereby playing a role in the process of community assembly.

Climatic factors have complex effects on community species diversity and PD. Studies have shown that precipitation can alleviate the negative impacts of drought, promote the recovery of annuals, and enhance the richness of perennial herbs, thereby increasing species diversity within communities [67,68]. In this study, PD, the Margalef index, and the Shannon–Wiener index were significantly negatively correlated with the mean temperature of the coldest quarter and precipitation seasonality, but were significantly positively correlated with precipitation of the wettest month (Figure 8). This result suggests that species richness and PD decreased under conditions of low temperatures and high precipitation fluctuations. Low temperatures and high variability in precipitation may increase environmental stress, allowing for only a few well-adapted species to survive in unstable environments, thereby reducing diversity [69]. Simpson’s index, which measures species dominance, was found to be significantly positively correlated with precipitation seasonality. Thus, precipitation instability may enhance the competitiveness of certain dominant species, affecting the evenness of the community.

Variance partitioning analysis (VPA) results (Figure 9) reveal that the Pielou and Simpson indices of species diversity were predominantly influenced by soil factors, indicating that soil physicochemical properties, such as soil nutrients and pH, exerted strong control over species evenness and dominance. By contrast, the Margalef and Shannon–Wiener indices were primarily affected by climatic factors, suggesting that climate exerted a major influence on species diversity and abundance within communities, potentially playing a crucial role in species reproduction and distribution [70]. PD and its structure were also largely shaped by climatic factors, implying that long-term climatic conditions may determine species evolution and adaptive selection. These findings were consistent with those of Zheng et al. [56] regarding shrub communities in the arid and semi-arid regions of the Mongolian Plateau. Overall, climatic factors play a significant role in regulating community diversity and phylogenetic structure, particularly under extreme and fluctuating climatic conditions, which exert strong filtering effects on communities. The combined influence of soil and climatic factors is crucial in maintaining plant community diversity and ecological adaptation, especially in arid and semi-arid regions.

## 4. Materials and Methods

### 4.1. Study Site

**Figure 10:**
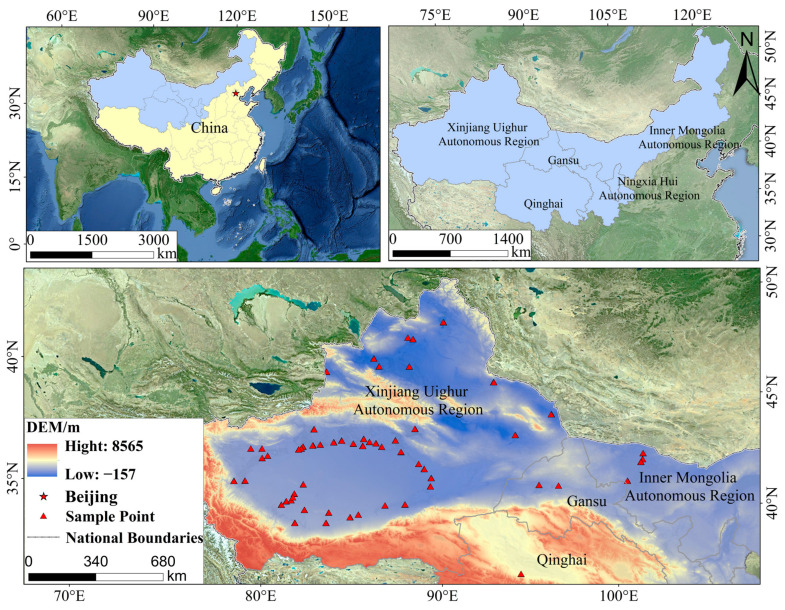
Study area and sampling sites.

This study was conducted in the *P. euphratica* distribution regions of Northwest China (30–50° N, 74–110° E), encompassing the provinces of Xinjiang, Inner Mongolia, Gansu, and Qinghai (Figure 10). The northwestern region spans a vast area from east to west, with altitudes ranging from −157 m to 8565 m, and features a highly complex geographical environment. Located deep within the Eurasian continent, the region is minimally affected by oceanic monsoons and is predominantly characterised by arid and semi-arid climate conditions. The region experiences harsh winters characterised by extreme cold and dryness, while summers are notably hot. Precipitation is unevenly distributed, exhibiting a clear decreasing gradient from east to west, ranging from a maximum of approximately 400 mm to a minimum of less than 50 mm [71,72]. The average annual precipitation is around 150 mm, whereas average annual evaporation exceeds 2500 mm. The region experiences significant diurnal temperature fluctuations, prolonged sunshine, and peak solar radiation between May and July [73]. Over the past 50 years, temperatures in Northwest China have shown fluctuations, generally trending upward, and a rate of temperature change of about 0.49 °C/10a [74,75].

Most of the rivers in the study area are inland rivers, which are significantly influenced by snow and ice melt and mountain precipitation. Moreover, they are predominantly seasonal. Among them, the Tarim River is the longest inland river in China, and it serves as the primary water source sustaining the growth of desert vegetation in the Tarim Basin [76]. The soil in this region is relatively infertile, supporting a landscape primarily dominated by desert flora, including *Populus euphratica*, *Populus pruinosa*, *Tamarix chinensis*, *Alhagi camelorum*, and *Haloxylon ammodendron*. It is mainly distributed in the Hexi Corridor, western Inner Mongolia, and among the basins.

### 4.2. Sample Plot Survey and Data Collection

In 2023, a botanical survey was conducted in the primary distribution area of *P. euphratica* in Northwest China. A total of 58 *P. euphratica* forest sample plots, each covering an area of approximately 1 km × 1 km, were established. Within each plot, nine 50 m × 50 m subplots were set up for repeated measurements, resulting in a total of 522 subplots. The distance between sample plots was maintained between 300 and 500 m. For each subplot, data were recorded on latitude, longitude, species composition, abundance, frequency, diameter at breast height (measured at 130 cm for trees), basal diameter (for shrubs), and vegetation cover.

To calculate the soil data, we selected three soil sampling points diagonally from each 50 m × 50 m sample plot. Soil sample were collected at five depth intervals (0–20, 20–40, 40–60, 60–80, and 80–100 cm) at 20 cm increments. Soil samples collected from three different locations were combined into a single composite sample for each depth point. These composite samples were sieved and processed to measure various soil physicochemical properties, including soil moisture content (SMC), pH, electrical conductivity (EC), soil organic carbon (SOC), total salt (TS), total potassium (TK), total nitrogen (TN), and total phosphorus (TP). SMC was determined using the gravimetric method (aluminium box drying). Soil pH and conductivity were measured by using a pH meter and conductivity meter, respectively, on soil leachates. SOC content was assessed by the potassium dichromate oxidation method (external heating). TS content was determined using the residue drying method. TP was analysed using the sodium hydroxide fusion–ultraviolet spectrophotometric method. TK was measured by the sodium hydroxide extraction–flame photometric method (INESA, FP4231, Shanghai, China), and TN was quantified using a Kjeldahl method (Hanon, K9840, Shanghai, China).

The climate data used in this study were derived from historical monthly records in the WorldClim database (https://worldclim.org/, accessed on 24 May 2024). The dataset included monthly maximum and minimum temperatures, as well as monthly precipitation, covering the period from 2001 to 2020. These data were processed using dismo package in R 4.4.1, with a spatial resolution of 2.5 arc minutes.

### 4.3. Calculation of Species Diversity Indices

The importance value (IV) is a quantitative indicator of species dominance within a community. Using the IV matrix, sample plots in the study area were classified with the ‘Twinspan’ package in R. The formula for calculating the IV for each species in a sample plot is as follows:

(1) $${IV}_{tree}=\frac{RA+RF+RD}{3},$$

(2) $${IV}_{shrubandherb}=\frac{RA+RF+RC}{3},$$

where RA represents relative abundance, calculated as the number of individuals of a species in the sample divided by the total number of plants of individuals of all species in the sample. RF denotes relative frequency, calculated as the frequency of occurrence of a species divided by the total frequency of all species in the sample. RD indicates relative dominance, measured as the sum of the diameter at breast height (DbH) area for a species divided by the total DbH area of all species in the sample. RC refers to relative cover, calculated as the total cover area of a species divided by the total cover of all species in the sample.

Species diversity provides insight into aspects such as the evenness and richness of plant species. In this study, the Shannon–Wiener diversity index, Simpson dominance index, Pielou evenness index, and Margalef richness index were used to characterise the α-diversity of species at each sample site. The calculation formulas are as follows:

Shannon–Wiener Diversity Index [77]:

(3) $$H=-\sum_{i=1}^{S}P_{i}\ln p_{i},$$

Simpson Dominance Index [78]:

(4) $$C=\sum_{i=1}^{S}P_{i}^{2},$$

Pielou Evenness Index [79]:

(5) $$J=\frac{H}{\ln S},$$

Margalef Richness Index [80]:

(6) $$R=\frac{S-1}{\ln N},$$

where S represents the number of species in the sample, Pi is the proportion of the importance value of species i relative to the total importance value of the sample, and N denotes the total number of individuals of all species in the sample.

### 4.4. Construction of the Phylogenetic Tree and Calculation of Phylogenetic Indices

The APG Ⅲ classification system within the plantlist package in R was used to perform a batch query for 107 species, generating a comprehensive list of families, genera, and species. Phylogenetic sequences were obtained using the V.PhyloMaker package [81], and phylogenetic trees were constructed with FigTree.v1.4.4 software [25].

On the basis of the constructed phylogenetic tree, phylogenetic indices of plant communities were calculated. In this study, Faith’s [82] PD index was used to represent the community’s phylogenetic diversity (PD), defined as the total sum of the evolutionary branch lengths of plant species on the phylogenetic tree. The net relatedness index (NRI) and nearest taxon index (NTI) were employed to characterise the phylogenetic structure. NRI represents the standardised effect size of the mean phylogenetic distance (MPD), while NTI reflects the mean nearest phylogenetic distance (MNTD) between species within the community. In this study, Model 2 in PHYLOCOM was selected, where species at each sampling point were randomly drawn from the phylogenetic pool. When NRI and NTI are greater than 0, the phylogenetic structure is considered aggregated, indicating that the community consists of closely related species. Conversely, when NRI and NTI are less than 0, the phylogenetic structure tends to be dispersed, signifying that the community consists of distantly related species. If NRI and NTI are both equal to 0, the phylogenetic structure is random, suggesting the dominance of neutral processes [83]. The formulas for calculating NRI and NTI are as follows:

(7) $$NRI=-1\times \frac{{MPD}_{sample}-{MPD}_{rndsample}}{{sdMPD}_{rndsample}},$$

(8) $$NTI=-1\times \frac{{MNTD}_{sample}-{MNTD}_{rndsample}}{{sdMNTD}_{rndsample}}$$

where MPD_sample_ and MNTD_sample_ represent the observed values in the sample, MPD_rndsample_ and MNTD_rndsample_ are the predicted values obtained from 999 random simulations under the null model, and sd is the standard deviation.

### 4.5. Data Analysis

In October 2023, all surveyed species were identified, and species names were confirmed. The data were compiled using Excel, and species diversity indices, including the Shannon–Wiener diversity index, Simpson dominance index, Pielou evenness index, and Margalef richness index, were calculated to assess species richness and evenness. All identified species were classified into families using the APG III classification system. Phylogenetic trees were constructed using the V.PhyloMaker package, and PD, NRI, and NTI were calculated in R. One-way ANOVA was conducted using Origin to compare species diversity, PD, and phylogenetic structure across different community groups to assess whether significant differences exist between community types. Linear regression was performed between PD and species richness (SR) to analyse their correlation. Additionally, a one-sample *t*-test was applied to NRI and NTI values to determine whether their means significantly deviate from zero. If the mean value was significantly greater or less than zero, then the community’s phylogenetic structure was either clustered or dispersed, respectively.

We conducted principal component analysis (PCA; with more than 80% explanation) on 8 soil factors and 19 climate factors, retaining components that explained more than 80% of the variance. Analysis of covariance was performed to address multicollinearity, and climate and soil factors with variance inflation factors (VIFs) > 10 were eliminated [84]. Pearson correlation analyses were employed in Origin to examine the relationships between indices and environmental factors, with significance levels set at between 0.05 and 0.01. The unique and shared effects of soil and climate were visualised using Venn diagrams generated through variance partitioning analysis (VPA) with the Vegan package in R.

## 5. Conclusions

Uncovering the processes and mechanisms underlying forest biodiversity has become a focal area of research in community ecology, yet studies on poplar communities in arid and semi-arid regions remain relatively limited. This study aimed to investigate the species diversity of *P. euphratica* communities, examine their community assembly processes and analyse the main factors influencing these dynamics. The results reveal that species diversity across different types of *P. euphratica* communities was low, with a significant correlation between species diversity and PD. Among these, the *P. euphratica*–*H. ammodendron* association group (Group 4) exhibited the highest levels of species and PD, identifying it as a key community that should be prioritised for conservation. From an evolutionary perspective, *P. euphratica* communities in Northwest China have undergone an extensive evolutionary process, demonstrating niche conservation in response to environmental changes, with competitive exclusion emerging as the predominant mechanism influencing community assembly. By analysing the effects of environmental factors on species and PD, we found that the key factors driving *P. euphratica* communities in Northwest China included TP, pH, SMC, and TK, as well as the mean temperature of the coldest quarter (BIO11), precipitation of the wettest month (BIO13) and precipitation seasonality (BIO15). The degree of evenness of species distribution and the growth of individual dominant species were primarily influenced by soil factors, whereas the species richness was mainly affected by climatic factors. The degree of phylogeny among species were also significantly influenced by climatic factors.

In summary, elucidating the assembly mechanism of *P. euphratica* communities from an evolutionary perspective offers a novel approach to understanding these ecosystems. The findings of this study provide valuable insights into the community assembly processes of *P. euphratica* and serve as a scientific basis for the ecological restoration of desert riparian forests and the conservation of biodiversity in Northwest China. Although this study focused on species diversity and phylogenetic relationships, it did not incorporate functional trait analysis or fully account for the influence of geographical factors and groundwater on *P. euphratica* communities. Future research should place strong emphasis on functional trait analyses and consider a broad range of environmental factors to comprehensively uncover the ecological processes driving the assembly of *P. euphratica* communities.

## Figures and Tables

**Table 1:** *t*-tests for NRI and NTI means of 0 for different community types.

	Cluster	Mean of NRI/NTI	Standard Deviation	*t*	*p*
NRI	Group 1	−0.8908	0.5747	−5.589	0.000
	Group 2	−0.5503	0.5690	−5.639	0.000
	Group 3	−1.2270	0.2686	−9.138	0.003
	Group 4	0.2355	2.0923	0.298	0.776
NTI	Group 1	0.2874	0.8420	1.231	0.242
	Group 2	−0.4505	0.9606	−2.735	0.010
	Group 3	−1.6500	1.4278	−2.311	0.104
	Group 4	−0.8541	1.6748	−1.349	0.226

## Data Availability

The data presented in this study are available on request from the corresponding author.

## Supplementary Materials

The following supporting information can be downloaded at: https://www.mdpi.com/article/10.3390/plants13233283/s1, Table S1. Plant species and related families in the sampling plots; Table S2. Comparison table of 19 climate factors.
