# Supplementary material for: Community Assembly Mechanisms of Populus euphratica in Northwest China and Their Relationship with Environmental Factors
Source: Plants (Basel). 2024 Nov 22;13(23):3283. doi: 10.3390/plants13233283 (PMC11644819; doi:10.3390/plants13233283)
Supplement: Supplementary file 1 [file plants-13-03283-s001.zip › plants-3291109-supplementary.pdf]

Supplementary files

Table S1. Plant species and related families in the sampling plots.

No.	Species name	Family	Genus
1	*Achnatherum splendens*	Poaceae	*Achnatherum*
2	*Acroptilon repens*	Asteraceae	*Acroptilon*
3	*Aeluropus pungens*	Poaceae	*Aeluropus*
4	*Alhagi sparsifolia*	Fabaceae	*Alhagi*
5	*Anabasis aphylla*	Amaranthaceae	*Anabasis*
6	*Artemisia argyi*	Asteraceae	*Artemisia*
7	*Artemisia blepharolepis*	Asteraceae	*Artemisia*
8	*Artemisia desertorum*	Asteraceae	*Artemisia*
9	*Artemisia frigida*	Asteraceae	*Artemisia*
10	*Artemisia lavandulaefolia*	Asteraceae	*Artemisia*
11	*Arthrophytum iliense*	Amaranthaceae	*Arthrophytum*
12	*Asparagus persicus*	Asparagaceae	*Asparagus*
13	*Bassia dasyphylla*	Amaranthaceae	*Bassia*
14	*Berberis kaschgarica*	Berberidaceae	*Berberis*
15	*Bromus inermis*	Poaceae	*Bromus*
16	*Calamagrostis epigeios*	Poaceae	*Calamagrostis*
17	*Calamagrostis pseudaphragmites*	Poaceae	*Calamagrostis*
18	*Calligonum roborovskii*	Polygonaceae	*Calligonum*
19	*Centaurium pulchellum*	Gentianaceae	*Centaurium*
20	*Ceratocarpus arenarius*	Amaranthaceae	*Ceratocarpus*
21	*Ceratoides latens*	Amaranthaceae	*Ceratoides*
22	*Chenopodium acuminatum*	Amaranthaceae	*Chenopodium*
23	*Chenopodium album*	Amaranthaceae	*Chenopodium*
24	*Chenopodium ficifolium*	Amaranthaceae	*Chenopodium*
25	*Chenopodium glaucum*	Amaranthaceae	*Chenopodium*
26	*Chenopodium rubrum*	Amaranthaceae	*Chenopodium*
27	*Chenopodium urbicum*	Amaranthaceae	*Chenopodium*
28	*Cirsium setosum*	Asteraceae	*Cirsium*
29	*Clematis orientalis*	Ranunculaceae	*Clematis*
30	*Conyza canadensis*	Asteraceae	*Conyza*
31	*Cynanchum sibiricum*	Apocynaceae	*Cynanchum*
32	*Diarthron linifolium*	Thymelaeaceae	*Diarthron*
33	*Dodartia orientalis*	Mazaceae	*Dodartia*
34	*Elaeagnus angustifolia*	Elaeagnaceae	*Elaeagnus*
35	*Glycyrrhiza glabra*	Fabaceae	*Glycyrrhiza*
36	*Glycyrrhiza inflata*	Fabaceae	*Glycyrrhiza*
37	*Glycyrrhiza uralensis*	Fabaceae	*Glycyrrhiza*
38	*Gueldenstaedtia verna*	Fabaceae	*Gueldenstaedtia*
39	*Halimodendron halodendron*	Fabaceae	*Halimodendron*
40	*Halocnemum strobilaceum*	Amaranthaceae	*Halocnemum*
41	*Halogeton arachnoideus*	Amaranthaceae	*Halogeton*
42	*Halogeton glomeratus*	Amaranthaceae	*Halogeton*
43	*Halostachys caspica*	Amaranthaceae	*Halostachys*
44	*Haloxylon ammodendron*	Amaranthaceae	*Haloxylon*
45	*Heliotropium ellipticum*	Heliotropiaceae	*Heliotropium*
46	*Hexinia polydichotoma*	Asteraceae	*Hexinia*
47	*Inula salsoloides*	Asteraceae	*Inula*
48	*Iris lactea var. chinensis*	Iridaceae	*Iris*
49	*Kalidium foliatum*	Amaranthaceae	*Kalidium*
50	*Kalidium schrenkianum*	Amaranthaceae	*Kalidium*
51	*Karelinia caspia*	Asteraceae	*Karelinia*
52	*Kochia melanoptera*	Amaranthaceae	*Kochia*

53	*Kochia prostrata*	Amaranthaceae	*Kochia*
54	*Lepidium latifolium*	Brassicaceae	*Lepidium*
55	*Leymus chinensis*	Poaceae	*Leymus*
56	*Leymus secalinus*	Poaceae	*Leymus*
57	*Leymus yiunensis*	Poaceae	*Leymus*
58	*Limonium bicolor*	Plumbaginaceae	*Limonium*
59	*Limonium kaschgaricum*	Plumbaginaceae	*Limonium*
60	*Lotus corniculatus*	Fabaceae	*Lotus*
61	*Lycium chinense*	Solanaceae	*Lycium*
62	*Lycium ruthenicum*	Solanaceae	*Lycium*
63	*Medicago lupulina*	Fabaceae	*Medicago*
64	*Mulgedium tataricum*	Asteraceae	*Mulgedium*
65	*Myricaria bracteata*	Tamaricaceae	*Myricaria*
66	*Nitraria sibirica*	Nitrariaceae	*Nitraria*
67	*Nitraria tangutorum*	Nitrariaceae	*Nitraria*
68	*Onopordum acanthium*	Asteraceae	*Onopordum*
69	*Oxytropis fetissovii*	Fabaceae	*Oxytropis*
70	*Oxytropis glabra*	Fabaceae	*Oxytropis*
71	*Peganum harmala*	Nitrariaceae	*Peganum*
72	*Phragmites australis*	Poaceae	*Phragmites*
73	*Plantago depressa*	Plantaginaceae	*Plantago*
74	*Poacynum pictum*	Apocynaceae	*Poacynum*
75	*Populus alba var. pyramidalis*	Salicaceae	*Populus*
76	*Populus euphratica*	Salicaceae	*Populus*
77	*Populus pruinosa*	Salicaceae	*Populus*
78	*Potentilla multifida*	Rosaceae	*Potentilla*
79	*Reaumuria kaschgarica*	Tamaricaceae	*Reaumuria*
80	*Reaumuria songarica*	Tamaricaceae	*Reaumuria*
81	*Salicornia europaea*	Amaranthaceae	*Salicornia*
82	*Salsola collina*	Amaranthaceae	*Salsola*
83	*Salsola lanafa*	Amaranthaceae	*Salsola*
84	*Salsola nitraria*	Amaranthaceae	*Salsola*
85	*Salsola passerina*	Amaranthaceae	*Salsola*
86	*Salsola ruthenica*	Amaranthaceae	*Salsola*
87	*Salsola soda*	Amaranthaceae	*Salsola*
88	*Scorzonera mongolica*	Asteraceae	*Scorzonera*
89	*Sonchus oleraceus*	Asteraceae	*Sonchus*
90	*Sophora alopecuroides*	Fabaceae	*Sophora*
91	*Sphaerophysa salsula*	Fabaceae	*Sphaerophysa*
92	*Suaeda acuminata*	Amaranthaceae	*Suaeda*
93	*Suaeda glauca*	Amaranthaceae	*Suaeda*
94	*Suaeda heterophylla*	Amaranthaceae	*Suaeda*
95	*Suaeda linifolia*	Amaranthaceae	*Suaeda*
96	*Suaeda salsa*	Amaranthaceae	*Suaeda*
97	*Suaeda stellatiflora*	Amaranthaceae	*Suaeda*
98	*Tamarix chinensis*	Tamaricaceae	*Tamarix*
99	*Tamarix hispida*	Tamaricaceae	*Tamarix*
100	*Tamarix leptostachys*	Tamaricaceae	*Tamarix*
101	*Tamarix ramosissima*	Tamaricaceae	*Tamarix*
102	*Tamarix taklamakanensis*	Tamaricaceae	*Tamarix*
103	*Taraxacum mongolicum*	Asteraceae	*Taraxacum*
104	*Taraxacum stenolobum*	Asteraceae	*Taraxacum*
105	*Tribulus terrester*	Zygophyllaceae	*Tribulus*
106	*Xanthium sibiricum*	Asteraceae	*Xanthium*
107	*Zygophyllum fabago*	Zygophyllaceae	*Zygophyllum*

Table S2. Comparison table of 19 climate factors.

No.	Climatic factor
BIO1	Annual Mean Temperature
BIO2	Mean Diurnal Range (Mean of monthly (max temp - min temp))
BIO3	Isothermality (BIO2/BIO7) (×100)
BIO4	Temperature Seasonality (standard deviation ×100)
BIO5	Max Temperature of Warmest Month
BIO6	Min Temperature of Coldest Month
BIO7	Temperature Annual Range (BIO5-BIO6)
BIO8	Mean Temperature of Wettest Quarter
BIO9	Mean Temperature of Driest Quarter
BIO10	Mean Temperature of Warmest Quarter
BIO11	Mean Temperature of Coldest Quarter
BIO12	Annual Precipitation
BIO13	Precipitation of Wettest Month
BIO14	Precipitation of Driest Month
BIO15	Precipitation Seasonality (Coefficient of Variation)
BIO16	Precipitation of Wettest Quarter
BIO17	Precipitation of Driest Quarter
BIO18	Precipitation of Warmest Quarter
BIO19	Precipitation of Coldest Quarter
